# Targeting maladaptive overcontrol with radically open dialectical behaviour therapy in a day programme for adolescents with restrictive eating disorders: an uncontrolled case series

**DOI:** 10.1186/s40337-020-00338-9

**Published:** 2020-11-13

**Authors:** Julian Baudinet, Mima Simic, Helena Griffiths, Cecily Donnelly, Catherine Stewart, Elizabeth Goddard

**Affiliations:** Maudsley Centre for Child and Adolescent Eating Disorders, Maudsley Hospital, De Crespigny Park, Denmark Hill, London, SE8 5AZ UK

**Keywords:** RO-DBT, Eating disorders, Anorexia nervosa, Day programme, Partial hospitalization programme

## Abstract

**Background:**

Radically Open Dialectical Behaviour Therapy (RO-DBT) was developed to target maladaptive overcontrol, a proposed core difficulty of restrictive eating disorders. RO-DBT is now the main group treatment model at the Intensive day Treatment Programme (ITP), Maudsley Hospital. This ITP case series aimed to investigate whether overcontrol is associated with restrictive eating disorder symptoms in adolescents and to evaluate ITP outcomes since RO-DBT skills classes were introduced.

**Method:**

Self-report measures of eating disorder symptoms and temperament, personality and social characteristics linked to overcontrol were collected at assessment and discharge from ITP for all consecutive adolescents who attended between February 2015 and January 2019 (*N* = 131). Weight change, global outcomes and treatment needs post-ITP were also recorded.

**Results:**

Eating disorder symptoms at assessment were significantly correlated with overcontrol factors, including social connectedness (*r* = −.67), reward responsivity (*r* = −.54)*,* and cognitive inflexibility (*r* = .52). Adolescents stayed in ITP on average 13.40 weeks. 70.8% had a Good-Intermediate outcome on Morgan-Russell scale. 4.6% did not respond and were referred to inpatient treatment from ITP. Significant improvements in drive for thinness (*d* = .33), depressive mood (*d* = .41), social connectedness (*d* = .48), and emotional expressiveness (*d* = .97) were reported at discharge. No changes were observed in perfectionism or negative temperament.

**Conclusions:**

This study offers preliminary evidence that eating disorder symptoms are associated with overcontrol factors in adolescence and that they can improve with RO-DBT informed day programme treatment. RO-DBT is a promising treatment that offers a new way of conceptualising treatment targets and recovery for adolescent restrictive eating disorders.

## Plain English summary

Restrictive eating disorders have been associated with overcontrolled coping, e.g. high levels of perfectionism, inflexibility, and a tendency to inhibit emotional expression leading to loneliness and social isolation. Radically Open Dialectical Behaviour Therapy (RO–DBT) suggests that targeting this coping style will lead to improvements in eating disorder symptoms. RO-DBT proposes that overcontrol is the main difficulty, not the eating disorder symptoms themselves. This study examined the relationship between overcontrol and adolescent eating disorder symptoms. RO-DBT skills classes were included in a day programme and changes in overcontrolled coping and eating disorder symptoms at the end of treatment were measured. Results from 131 adolescents with restrictive eating disorders showed that more severe eating disorder symptoms co-occurred with more overcontrol on some measures (social connection, reward sensitivity, and flexibility). Only 4.6% did not improve during the treatment and were referred for inpatient treatment; a low proportion compared to other day programme studies. Adolescents also reported significant improvements in drive for thinness, depressed mood, social connectedness, and emotional expression at discharge. These results suggest that a day hospital programme that includes RO-DBT can have a positive impact on overcontrolled coping and the clinical outcomes of adolescent restrictive eating disorders.

## Background

Outpatient family therapies for adolescents with restrictive eating disorders have moderate to good outcomes. However, it is now well established that there is a significant proportion for whom family therapy is not enough [1, 2]. Historically, this group of young people were offered inpatient treatment, which can be lengthy and costly, with poor or mixed outcomes [3, 4]. To try and better support this group of young people and their families, day programmes, or partial hospitalisation programs, have been developed as an alternative intensive treatment option.

Research investigating the efficacy of day programmes for eating disorders has predominately focused on adults [5, 6], however there is a growing body of evidence for day programmes for adolescents [7–13], including one randomised controlled trial [14]. Results demonstrate that day programme attendance leads to significant improvements in physical and psychological health [8–10, 12, 13, 15–19], which are maintained at follow up [7, 8].

Despite these promising findings, no day programme is effective for all adolescents, with some going on to require inpatient care and/or multiple day programme admission. Dancyger et al. [20] reported that 26% of adolescents left their day programme before goals were met and 15% required inpatient treatment. Similarly, Simic et al. [8] reported that 18% needed to be discharged from day programme to inpatient treatment. Clearly there is a need to continue to improve day programme treatments.

In recent years, new evidence is emerging of a distinct bio-temperamental profile for people with restrictive eating disorders [21–23]. This bio-temperamental profile is characterised by high levels of neuroticism [24], perfectionism [25], obsessionality [26], persistence [22], and low reward sensitivity [27, 28]. Socially, people with restrictive eating disorders report distant relationships and high levels of loneliness [29] as well as high suppression of negative emotions and a minimisation of their own needs in order to preserve their relationships [30]. Hempel et al. [21] propose that this cluster of traits and difficulties associated with restrictive eating disorders point to a core difficulty with maladaptive self-control, or overcontrol.

Concepts of overcontrol and undercontrol have been described in the literature in various form for some time [31–34]. The current conceptualisation of maladaptive overcontrol is defined by four core difficulties; a) low openness and tolerance of uncertainty b) cognitive and behavioural rigidity and inflexibility, c) pervasive inhibited emotional expression and emotional awareness, and d) low social connectedness and intimacy which is often manifested in aloof and distant relationships [35]. Other markers of overcontrol include low reward sensitivity, high threat sensitivity, high inhibitory control and high attention to detail [35, 36]. Conversely, undercontrol is characterised by low inhibitory control (e.g. impulsivity, disinhibition, novelty seeking, affective lability) and a more chaotic pattern of relationships [35, 36]. Overcontrol has been associated with internalising disorders, whereas undercontrol with externalising disorders [37].

Given the reconceptualization of restrictive eating disorders as primarily disorders of overcontrol [21], it has been proposed that treatments specifically targeting overcontrol may lead to improved outcomes, as they target the broader core psychopathology of eating disorders, which may also improve relapse rates, behaviours and cognitions. There is a need to examine the efficacy of treatments designed to target maladaptive overcontrol and the key bio-temperamental factors associated with overcontrol among adolescents with anorexia nervosa and restrictive eating disorders.

Radically Open Dialectical Behaviour Therapy (RO-DBT) is a new, trans-diagnostic, manualised treatment that targets psychological disorders associated with maladaptive overcontrol [35]. While traditional Dialectical Behaviour Therapy (DBT) [38] targets behaviours associated with maladaptive undercontrol, RO-DBT addresses maladaptive overcontrol via the novel change mechanism of targeting social connection and social signalling. RO-DBT posits that improving social signalling, the verbal and non-verbal cues that people constantly consciously and unconsciously send to others, will increase the closeness and quality of relationships and networks [35]. RO-DBT hypothesises that the development of closer social connections leads to improved eating disorder symptom management and reduction. Similar to standard Dialectical Behaviour Therapy, RO-DBT is offered as a combination of weekly groups (named skills classes in RO-DBT) and concurrent weekly individual therapy sessions [35]. There is emerging evidence that it is effective for adults with eating disorders [39] and the model has recently been adapted for adolescents [40].

In an attempt to improve outcomes of day programme treatment for adolescents with restrictive eating disorders, the clinical team at the Intensive Treatment Programme (ITP), a day programme at the Maudsley Centre for Child and Adolescent Eating Disorders (MCCAED), introduced RO-DBT skills classes into the programme. All ITP staff were trained in the model and RO-DBT skills classes became the main group therapy intervention offered from March 2015.

This study has two aims. Firstly, to explore whether temperament, personality and coping related to overcontrol are associated with restrictive eating disorder and the related psychopathology in adolescence. Secondly, to evaluate whether ITP with RO-DBT skills classes leads to changes in these characteristics as well as in eating disorder symptoms and psychological symptoms associated with eating disorders. Given the exploratory nature of this uncontrolled case series, no a priori hypotheses were generated.

## Method

### ITP Programme description

ITP is a day programme that operates Monday to Friday at The Maudsley Hospital, London, UK. Included in the therapeutic treatment programme are individual, family and group therapy, meal support and education support. ITP is a rolling open-ended programme aiming to establish a stable trajectory towards recovery and effective re-engagement with outpatient treatment. The group therapy programme is spread over 6.25 h per week, of which 2.5 h are dedicated to RO-DBT skill classes, split over 2 sessions per week. RO-DBT skill class content was modified from Lynch’s original skills manuals [41] to be more adolescent appropriate and offered over 16 sessions, rather than the original 30. The content of the skill classes is repeated approximately every 8 weeks. The remaining ITP group therapy content includes cognitive behavioural therapy (CBT) (1.5 h), cognitive remediation treatment (CRT) (45 min) and art therapy (1 h).

### Study design

Adolescents completed a diagnostic interview at assessment, as well as self-report questionnaires at assessment and discharge from ITP. The battery of self-report measures targeted three main domains (a) eating disorder symptoms and psychological symptoms associated with eating disorders (mental health symptomatology), (b) temperamental, personality and coping factors associated with overcontrol, and (c) relationship quality and attachment. Time one assessment measures were used to explore the relationship between these three domains and eating disorder symptoms. Changes on these measures at discharge from ITP were used to evaluate whether ITP was effective in supporting reductions in eating disorder symptoms as well as overcontrolled coping and related domains such as social connectedness. The battery of measures was changed twice with additional measures added at two different time points to better evaluate ITP outcomes and the assessment of factors associated with overcontrol. Changes in weight, menstruation status, length of treatment and treatment needs following ITP were also recorded.

### Measures

#### Self-report measures

##### Measures assessing eating disorder and other mental health symptomatology

Eating disorder symptoms and psychological symptoms associated with eating disorders were assessed using the Eating Disorder Inventory – 3rd Version (EDI-3) [42]. The EDI-3 consists of 91 items organised into 12 primary scales. Five composite scales and a global score can be derived from the 12 subscales, including an eating disorder risk composite. The EDI-3 is widely used, has good reliability, internal consistency and discriminate validity [42, 43] and has published adolescent norms [42].

Symptoms of depression were screened using the 33-item Moods and Feelings Questionnaire (MFQ) [44]. The MFQ is a reliable measure of depression in children and adolescents. Scores of 27 and higher indicate the presence of depression [45, 46]. The MFQ been shown to have good validity, reliability and internal consistency with adolescents [47].

At assessment all adolescents and one or both parent also completed the online version of the Development and Wellbeing Assessment (DAWBA, [48]) to assess symptoms of comorbid psychiatric diagnoses. The DAWBA is a widely used structured diagnostic assessment that can generate DSM-V [49] and ICD-10 [50] psychiatric diagnoses for two to 17-year olds [48]. The DAWBA involves two steps. Firstly, computer-generated predicted diagnoses are provided from self-report responses to an online questionnaire. These responses can then be reviewed by an expert clinical rater to confirm individual diagnoses. The computer-generated predicted diagnoses alone have been shown to be a useful tool to estimate comorbidity for children and adolescent [51], although may slightly underestimates total rates of psychiatric disorders in this group. In this study, only the predicted diagnoses are reported.

##### Measures assessing temperament, personality factors and coping associated with overcontrol

Measures targeting factors associated with overcontrol were selected to identify temperament, personality and coping factors for adolescents with restrictive eating disorder. As overcontrol is a recently defined construct not previously investigated with adolescents, validated adolescent measures were not available for the full range of overcontrol related factors at the time of the study design. In their absence, adult measures were used.

The Negative Temperament subscale of the Schedule of Non-adaptive and Adaptive Personality for Youth (SNAPY-Y) [52] was used to assess level of maladaptive negative temperament and its stability across treatment. This subscale measures tendencies towards fear, anger, sadness, irritability and distress. Given temperament is relatively stable and likely biologically based [53], change was not expected on the SNAP-Y from baseline to discharge. The SNAP-Y has shown to be a valid measure of personality in adolescence, with good internal consistency and structural validity [52], as well as availability community and clinical norms [52, 54].

The Five Factor Obsessive Compulsive Inventory – Short Form (FFOCI-SF) [55, 56] was included to assess levels of risk aversion, cognitive flexibility, perfectionism, workaholism and punctiliousness. The FFOCI-SF has not been validated for children and adolescents, however, was included in this study in the absence of a validated measure of obsessive-compulsive personality in children and adolescence and recent developments indicating personality pathology does occur in children and adolescents [57]. The FFOCI-SF has demonstrated good discriminant validity and internal consistency with an undergraduate university sample [56].

Reward processing, as one marker of overcontrol, was measured using the Temporal Experience of Pleasures [58]. The 18-item self-report measure is based on Klein’s [59] model of anhedonia and has two subscales that measure distinct facets of trait-based reward processing; namely, the anticipatory (TEPS-ANT) and consummatory (TEPS-CON) aspects. The anticipation subscale (TEPS-ANT) assesses how much someone “wants” or is motivated by pleasurable things (example item: “when something exciting is coming up in my life, I really look forward to it”). The consummatory scale assesses how much a person “likes” or is able to enjoy pleasurable things in the moment (example item: “I love the sound of rain on the windows when I’m lying in my warm bed”). Anticipatory (“wanting”) aspects, as opposed to the consummatory (“liking”) aspects, of reward processing have been associated with motivation, reinforcement learning and reward-based decision-making [60]. The TEPS has not been used with an adolescent sample, however has demonstrates good internal consistency, test-retest reliability, and convergent and divergent validity in undergraduate university age samples [58].

The Emotion Regulation Questionnaire (ERQ) [61] is a validated 10-item scale designed to measure emotion regulation using two separate sub-scales, the tendency to regulate emotions via cognitive reappraisal strategies or via the suppression of emotion expression. Cognitive reappraisal strategies refer to when a person changes how they think in an attempt to change their emotional experience (example item: “when I want to feel less negative emotion [such as sadness or anger], I change what I’m thinking about”). The suppression of emotion expression subscale assesses a person’s attempts to inhibit the behavioural expression of their emotions (example item: “when I am feeling negative emotions, I make sure not to express them”) [62]. Cognitive reappraisal scores are typically considered adaptive and associated with low psychological distress and alexithymia, whereas expressive suppression is considered less adaptive and associated with psychological distress and alexithymia [62]. The ERQ has demonstrated good reliability and validity [61], good internal consistency [62], and has been used with adolescents [63].

##### Measures assessing relationships quality and attachment

Level of perceived social withdrawal and isolation was assessed using the 8-item Withdrawal subscale of the Youth Self-Report questionnaire (WS-YSR) [64]. The YSR is a widely used and validated self-report measure used to assess a range of problems in adolescents [64].

The 20-item Social Connectedness Scale (SCS-R) [65] was used to assess how connected one feels to others in their social environment. Low scores indicate low levels of social connection. This measure has not been validated with adolescents, however, has demonstrated good validity and internal consistency in adult samples [65].

Attachment characteristics and the quality of relationships were assessed using the 40-item attachment styles questionnaire (ASQ) [66]. The ASQ contains five subscales assessing different dimensions of attachment, including relationship confidence, need for approval, discomfort with closeness, pre-occupation and relationships as secondary. The ASQ has been used with adults and adolescents [67]. It has been shown to be valid and reliable, with good internal consistency [66, 68, 69].

#### Physical health and other markers of ITP outcome

Information on physical health and other markers of ITP outcome were extracted from medical records. Weight gain was used as the primary marker of treatment outcome. Weight was calculated as a percentage of median Body Mass Index (%mBMI) adjusting for age and gender. The modified Morgan-Russell Global Outcome Assessment Schedule [70, 71] was used to classify outcome at discharge. Good outcome indicates weight greater than 85%mBMI with menstruation or premenarchal (under the age of 15 and before the onset of the first period) and no bulimic symptoms. Intermediate outcome indicates weight greater than 85%mBMI without menstruation or bulimic symptoms averaging < 1 per week over the last month. Poor outcome indicates weight below 85%mBMI without menstruation or bulimic symptoms averaging ≥1 per week over the last month. Intensity of treatment required at discharge from ITP was identified as either outpatient or inpatient (either a psychiatric or medical stabilisation admission) and was also considered a marker of treatment outcome.

### Sample

The total sample comprised 131 adolescents (age 11–18 years) who consecutively attended ITP between February 2015 and January 2019. Data from the first admission to ITP was used for this study (2 admissions *n* = 11, 3 admissions *n* = 1). Nine adolescents (6.9%) attended for 5 days or less and were considered non-starters. One person had a diagnosis of bulimia nervosa and was excluded from further data analysis. No exclusions were made based on previous treatment, clinical severity, medication or comorbid diagnosis.

### Analysis plan

Individual and group mean scores on all self-report measures were obtained at assessment and discharge from ITP. To analyse relationships at baseline (Aim 1) Pearson’s r correlations were conducted between EDI-3 composite scales and measures assessing the biopsychosocial factors linked with maladaptive overcontrol. Due to poor internal consistency at assessment (Cronbach a < 0.70) three scales were removed and not included in further analysis. These were the FFOCI-SF Punctiliousness subscale (a = 0.68), ASQ Confidence subscale (a = 0.68) and the ERQ cognitive reappraisal subscale (a = 0.65). Paired t-tests were then conducted to analyse the mean difference in self-report measures from assessment to discharge. Due to the large number of tests, the Benjamini-Hochberg procedure (adjusted to <.05) [72] was applied to correct for multiple comparisons. Given the exploratory nature of this study both uncorrected and corrected data are reported.

As measures were introduced at different time points over a four year period in a busy clinical service, there were inconsistencies in data collection and differences in the number of measures collected at different time points. As a result, there are varying degrees of missing data for each measure included in this study, particularly for the paired EDI-3. To examine potential sampling bias, analyses were conducted to compare those who had completed each questionnaire to those who had not, across key demographic and clinical outcomes. Results showed that there were no differences between those who had completed the outcome measures at time 1 (assessment: EDI-3, SCS-R, MFQ, WS-YSR, ASQ, FFOCI-SF, SNAP-Y, ERQ, TEPS) compared to those who did not complete them on age, %mBMI at beginning of treatment or referral source (inpatient or outpatient). Further analyses were conducted to examine the impact of missing data at time 2 (discharge). There were no differences between those who completed time 2 measures and those who did not complete end of treatment measures on age, %mBMI at beginning of treatment, or referral source (outpatient or inpatient).

## Results

### Demographics

Demographic information including age, gender and referral source of adolescents at assessment are presented in Table 1. All participants identified as cis gendered.

**Table 1:** Demographic Information at assessment

Age	15.02 years (sd = 1.52, range = 11–18)
Gender	
- Female	94.62% (*n* = 123)
- Male	5.38% (*n* = 7)
Referral Source	
- Outpatient	86.92% (*n* = 113)
- Inpatient	13.08% (*n* = 17)
- *Paediatric Admission*	*5.38% (n = 7)*
- *Adolescent Psychiatric Unit*	*3.85% (n = 5)*
- *Specialist Eating Disorder Unit*	*3.85% (n = 5)*
Primary diagnosis at assessment	
- Anorexia nervosa	93.13% (*n* = 122)
- *Restrictive subtype*	*83.84% (n = 109)*
- *Binge-purge subtype*	*4.61% (n = 6)*
- *Atypical anorexia nervosa*	*5.38% (n = 7)*
- Other Specified Feeding and Eating Disorder (OSFED)	6.15% (*n* = 8)
Comorbid diagnoses predicted at assessment	
- Major Depressive Disorder	41.54% (*n* = 54)
- OCD	10.00% (n = 13)
- Anxiety disorder (> 1 diagnosed)	46.92% (*n* = 61)
- *Generalised Anxiety Disorder*	*36.15% (n = 47)*
- *Social Phobia*	*24.62% (n = 32)*
- *Separation Anxiety*	*6.15% (n = 8)*
- *PTSD*	*1.54% (n = 2)*
- *Panic Disorder*	*6.92% (n = 9)*
- *Specific Phobia*	*13.08% (n = 17)*
- *Agoraphobia*	*3.85% (n = 5)*
- ASD	*0.77% (n = 1)*
- Missing	*6.92% (n = 9)*
Number of comorbidities predicted at assessment	
- 1 or more	57.69% (*n* = 75)
- 2 or more	38.46% (*n* = 50)
- 3 or more	23.85% (*n* = 31)

*Abbreviations*: *OCD* obsessive compulsive disorder, *PTSD* post-traumatic stress disorder, *ASD* autism spectrum disorder

### Correlation analysis at assessment

Pearson’s correlations between EDI-3 composites and measures assessing overcontrolled temperament, personality and the quality of relationships at assessment are presented in Table 2. Almost all correlations were moderate to strong and significantly correlated at the *p* < 0.01 level. The EDI-3 Eating Disorder Risk composite had a strong negative correlation with social connectedness (SCS-R) and moderate negative correlation with anticipation of pleasure (TEPS-ANT), reflecting that those with more eating disorder risk felt less socially connected and had reduced anticipation of pleasure. Furthermore, Eating Disorder Risk composite was moderately positively correlated with the social withdrawal subscale of the YSR and all subscales of the ASQ, indicating those with increased eating disorder risk were more socially withdrawn and had more difficulty in their attachment relationships. Both the Risk Aversion and Cognitive Inflexibility subscales were significantly moderately correlated with eating disorder risk, meaning those with increase eating disorder risk were more risk averse and cognitively inflexible.

**Table 2:** Pearson’s r correlation matrix for self-report measures at assessment

	EDI-3 Composites					
	Eating Disorder Risk	Ineffectiveness	Interpersonal Problems	Affective Problems	Overcontrol	Global Psychological Maladjustment
Relationship quality and attachment						
SCS-R	−.67^a^	−.79^a^	−.79^a^	−.74^a^	−.57^a^	−.84^a^
ASQ Discomfort	.54^a^	.71^a^	.74^a^	.63^a^	.54^a^	.76^a^
ASQ Preoccupation	.47^a^	.50^a^	.36^a^	.39^a^	.46^a^	.52^a^
ASQ Relationships as secondary	.44^a^	.37^a^	.49^a^	.45^a^	.53^a^	.53^a^
ASQ Need for approval	.57^a^	.66^a^	.51^a^	.48^a^	.55^a^	.66^a^
YSR Withdrawal subscale	.52^a^	.66^a^	.71^a^	.63^a^	.44^a^	.71^a^
Overcontrolled temperament, personality and coping						
FFOCI-SF Risk aversion	.48^a^	.49^a^	.39^a^	.33^a^	.39^a^	.53^a^
FFOCI-SF Inflexible	.52^a^	.52^a^	.40^a^	.41^a^	.49^a^	.59^a^
FFOCI-SF Perfectionism	.17	.12	−.02	−.08	.30^b^	.10
FFOCI-SF Workaholism	.06	−.02	−.01	−.03	.26^b^	.04
SNAP-Y Negative Temperament	.12	.24^b^	.01	.14	.24	.18
TEPS-ANT	−.54^a^	−.53^a^	−.59^a^	−.54^a^	−.38^a^	−.57^a^
TEPS-CON	−.25^b^	−.23^b^	−.28^a^	−.22^b^	−.18	−.26^b^
ERQ Emotional Suppression	.36^a^	.30	.44^a^	.38^a^	.41^a^	.46^a^

*Abbreviations*: *EDI-3* Eating Disorder Inveentory-3, *SCS-R* Social Connectedness Scale-Revised, *ASQ* Attachment Style Questionnaire, *YSR* Youth Self Report, *FFOCI-SF* Five Factor Obsessive Compulsive Inventory-Short Form, *SNAP-Y* Schedule for Nonadaptive and Adaptive Personality for Youth, *TEPS-ANT* Temporal Experience of Pleasure Scale-Anticipatory, *TEPS-CON* Temporal Experience of Pleasure Scale-Consummatory, *ERQ* Emotion Regulation Questionnaire

^a^correlation significant at .01 level; ^b^correlation significance at .05 level

The Perfectionism and Workaholism subscales of the FFOCI-SF, however, were not significantly correlated with Eating Disorder Risk composite. The Negative Temperament subscale of the SNAPY-Y was only significantly correlated with the Ineffectiveness composite of the EDI-3. All measures, except for the Consummatory subscale of the TEPS (TEPS-CON), were significantly correlated with the Overcontrol Composite of the EDI-3.

### ITP outcomes

The mean length of stay in ITP was 13.40 weeks (sd = 5.91, range 1–30 weeks). Mean percent median BMI at assessment was 82.39% (sd = 8.46). This increased to a mean of 89.51% (sd = 8.59) at discharge. This corresponds to a mean gain of 7.19%mBMI during ITP, at a mean rate of 0.54%mBMI per week. At discharge, more than 70% (*n* = 92) of adolescents had a Good or Intermediate outcome using the Morgan-Russell Global Outcome measure (Good = 53.08%, *n* = 69; Intermediate = 17.69%, *n* = 23). The remainder had a Poor outcome (28.46%, *n* = 37) and 1 had missing data and could not be categorized. Six people (4.62%) required inpatient treatment during or after ITP (specialist eating disorder unit = 3.08%, *n* = 4; general adolescent psychiatric unit = 1.54%, *n* = 2).

### Changes in symptoms of eating disorders and depression

There was a significant reduction in self-report eating disorder and associated symptomatology on seven of the 12 subscales of the EDI-3 (see Table 3). Notably, the drive for thinness subscale reduced from the typical clinical to low clinical range, while most others remained within the typical clinical range [42]. Adolescents reported a significant reduction in symptoms of depression on the MFQ with medium effect size (*d* = .41) over the course of ITP. Individuals scoring above the cut-off indicating the likely presence of depression (total score > 27) [45] reduced from 71.4% at assessment to 62.8% at discharge. After adjusting for multiple comparisons using the Benjamini-Hochberg procedure (adjusted to <.05) changes on the MFQ, EDI-drive for thinness subscale, EDI-personal alienation subscale and the EDI-interoceptive deficits subscale remained significant.

**Table 3:** Mean scores at assessment and discharge from the Intensive Treatment Programme (ITP) on self-report measures

Measure (n attendees whilst measure in use)	Assessment mean (sd)	Discharge mean (sd)	Test statistic (paired n)	Effect size	*Cronbach alpha*	
					*Pre ITP*	*Post ITP*
Eating disorder and mental health symptomatology						
EDI-3 (*n* = 105)						
- Drive for Thinness raw score	18.31^c^ (8.45)	15.16^b^ (8.92)	*p* = 0.01*^,a^ (*n* = 62)	*d* = 0.33	.91	.93
- Bulimia raw score	2.63^b^ (2.91)	2.84^b^ (4.15)	*p* = 0.71 (*n* = 62)	*d* = 0.05	.78	.83
- Body dissatisfaction raw score	27.60^c^ (11.95)	24.34^c^ (11.72)	*p* = 0.051 (*n* = 62)	*d* = 0.25	.94	.93
- Low self-esteem raw score	14.50^c^ (6.93)	12.63^c^ (6.88)	*p* = 0.04* (*n* = 62)	*d* = 0.26	.91	.90
- Personal Alienation raw score	14.28^c^ (7.24)	11.44^c^ (6.93)	*p* = 0.003** (*n* = 61)	*d* = 0.39	.88	.85
- Interpersonal insecurity raw score	12.21^c^ (7.19)	10.60^c^ (8.04)	*p* = 0.048* (*n* = 62)	*d* = 0.26	.87	.86
- Interpersonal alienation raw score	10.19^c^ (6.46)	8.45^c^ (6.60)	*p* = 0.014* (*n* = 62)	*d* = 0.32	.86	.85
- Interceptive deficits raw score	14.82^c^ (8.76)	11.74^c^ (9.00)	*p* = 0.004** (*n* = 62)	*d* = 0.38	.90	.89
- Emotional dysregulation raw score	8.71^c^ (5.88)	7.97^c^ (6.96)	*p* = 0.43 (*n* = 62)	*d* = 0.10	.82	.75
- Perfectionism raw score	11.85^c^ (6.22)	11.19^c^ (6.14)	*p* = 0.37 (*n* = 48)	*d* = 0.13	.76	.82
- Ascetism raw score	11.81^c^ (7.05)	9.81^c^ (7.50)	*p* = 0.027* (*n* = 59)	*d* = 0.29	.84	.86
- Maturity Fears raw score	15.76^d^ (7.78)	14.71^d^ (8.52)	*p* = 0.19 (*n* = 59)	*d* = 0.17	.88	.90
MFQ (*n* = 51)	38.05^e^ (15.62)	30.84^e^ (18.47)	*p* = 0.01*^,a^ (*n* = 43)	*d* = 0.41	.94	.98
Relationship quality and attachment						
SCS-R (*n* = 81)	67.97 (21.43)	75.51 (22.98)	*p* < 0.001***^,a^ (*n* = 67)	*d* = 0.48	.95	.97
ASQ (*n* = 101)						
- Discomfort	42.32 (10.99)	38.65 (11.60)	*p* < 0.001***^,a^ (*n* = 81)	*d* = 0.43	.82	.93
- Preoccupation	30.02 (7.53)	31.44 (7.67)	*p* = 0.048* (*n* = 81)	*d* = 0.22	.80	.78
- Relationships as secondary	20.23 (6.31)	18.46 (7.54)	*p* = 0.006**^,a^ (*n* = 81)	*d* = 0.32	.80	.87
- Need for approval	32.48 (6.66)	31.69 (7.16)	*p* = 0.24 (*n* = 81)	*d* = 0.13	.84	.86
YSR Withdrawal subscale (*n* = 101)	8.75^g^ (4.24)	6.48^f^ (4.60)	*p* < 0.001***^,a^ (*n* = 81)	*d* = 0.54	.89	.90
Overcontrolled temperament, personality and coping						
FFOCI-SF (*n* = 81)						
- Risk aversion	12.12 (4.20)	12.15 (10.93)	*p* = 0.98 (*n* = 65)	*d* < 0.01	.85	.88
- Inflexible	11.91 (3.64)	10.83 (3.83)	*p* = 0.007**^,a^ (*n* = 65)	*d* = 0.34	.82	.89
- Perfectionism	15.02 (3.30)	14.91 (3.45)	*p* = 0.75 (*n* = 65)	*d* = 0.04	.78	.85
- Workaholism	12.32 (3.83)	12.25 (4.08)	*p* = 0.83 (*n* = 65)	*d* = 0.03	.87	.89
SNAP-Y Negative Temperament (*n* = 81)	30.47 (10.11)	30.08 (11.43)	*p* = 0.67 (*n* = 62)	*d* = 0.05	.86	.87
TEPS (*n* = 113)						
- Anticipatory (TEPS-ANT)	32.79 (8.93)	36.79 (9.92)	*p* < 0.001***^,a^ (*n* = 96)	*d* = 0.42	.80	.71
- Consummatory (TEPS-CON)	32.18 (7.03)	35.19 (7.54)	*p* < 0.001***^,a^ (*n* = 96)	*d* = 0.51	.71	.74
ERQ Expressive Suppression (*n* = 51)	19.50 (6.82)	14.02 (7.41)	*p* < 0.001***^,a^ (*n* = 42)	*d* = 0.97	.87	.93

n for each measure provided in table indicates the number of young people who attended ITP while that measure was being administered

*Abbreviations*: *EDI-3* Eating Disorder Inveentory-3, *SCS-R* Social Connectedness Scale-Revised, *ASQ* Attachment Style Questionnaire, *YSR* Youth Self Report, *FFOCI-SF* Five Factor Obsessive Compulsive Inventory-Short Form, *SNAP-Y* Schedule for Nonadaptive and Adaptive Personality for Youth, *TEPS-ANT* Temporal Experience of Pleasure Scale-Anticipatory, *TEPS-CON* Temporal Experience of Pleasure Scale-Consummatory, *ERQ* Emotion Regulation Questionnaire, *sd* standard deviation

*significant at *p* < 0.05 level; **significance at *p* < 0.01 level; ***significance at *p* < 0.001 level

^a^significant after Benjamini-Hochberg adjustment (<.05) for multiple comparisons

^b^Low clinical range; ^c^typical clinical range; ^d^elevated clinical range (Garner, 2004)

^e^above clinical cut off for depression (total score > 27; Wood, et al., 1995)

^f^one standard deviation above community norms (mean = 3.1, sd = 2.5; Achenbach & Rescorla, 2001)

^g^two standard deviation above community norms (mean = 3.1, sd = 2.5; Achenbach & Rescorla, 2001)

### Changes in Overcontrolled temperament, personality and coping

On the Emotion Regulation Questionnaire (ERQ) there was a significant reduction in the suppression of emotional expression as an emotion regulation strategy with large effect size (*d* = .97). There was also a significant decrease in cognitive inflexibility with a small effect size (*d* = .34) on the FFOCI-SF, however no changes were observed on any other subscale of the FFOCI-SF. Similarly, there was no significant difference between assessment and discharge in self report of negative treatment (SNAP-Y). Finally, analysis revealed significant differences between assessment and discharge on both subscales of the TEPS, reflecting an increase in reward responsiveness (TEPS-ANT; d = .42) and the consumption of pleasure (TEPS-CON; d = .51). All observed significant changes remained significant after correction for multiple comparisons using the Benjamini-Hochberg procedure (adjusted to <.05).

### Changes in relationship quality and attachment

There was a significant increase in means scores on the SCS-R with medium effect size (*d* = .48), indicating improved social connectedness by the end of ITP. Mean scores on the WS-YSR significantly reduced with medium effect size (*d* = .54), representing a reduction in self-report level of perceived isolation and social withdrawal. Similarly, on the ASQ there was a significant reduction with small to medium effect size in adolescents’ level of discomfort in close relationships (*d* = .43), as well as an increase in their preoccupation with relationships (*d* = .22) and a reduction in relationships being seen as secondary (*d* = .32). No significant change was observed on the need for approval subscale of the ASQ. After adjusting for multiple comparisons using the Benjamini-Hochberg procedure (adjusted to <.05) all significant changes remained significant, except for the ASQ-Preoccupation subscale.

## Discussion

This is the first study to explore whether the RO-DBT concept of overcontrol is applicable to adolescents with restrictive eating disorders. This exploratory study provides preliminary evidence that temperament, personality and social factors related to overcontrol may be associated with restrictive eating disorder and related psychopathology in adolescence. The results also indicate that a RO-DBT informed day programme may lead to improvements in eating disorder symptoms and several factors associated with overcontrol.

Adolescents in this study reported high levels of both eating disorder symptoms and other difficulties associated with maladaptive overcontrol. At assessments the majority of adolescents attending ITP presented with eating disorder and associated psychological symptoms on the EDI-3 within the typical clinical range with the exception of maturity fears subscale, which was in the elevated clinical range, and the bulimia subscale, which was in the low clinical range [42]. Comorbidity for this group was the norm, with more than half (57.69%) predicted to have one or more comorbid diagnosis and almost one quarter (23.85%) with three or more at assessment. Seventy percent of the adolescents scored above the cut-off indicative of depression on the MFQ at assessment [45], indicating low mood, irrespective of a diagnosis, was also common for this group. Perceived social withdrawal and tendencies towards negative temperament were also both very high with mean scores more than two standard deviations above adolescent community norms on the WS-YSR [64] and SNAP-Y [52] respectively.

Correlation analysis of self-report measures at assessment confirmed significant moderate to strong associations between eating disorder symptom severity and overcontrol factors. These findings imply an interplay between eating disorder symptoms, sense of isolation and withdrawal, use of emotional suppression in coping and quality of attachment relationships. Furthermore, they show the association between eating disorder symptoms and cognitive rigidity, risk aversion and reduced capacity of adolescents to anticipate or experience pleasure. Together this suggests that adolescents with restrictive eating disorders struggle with a broad range of difficulties, many of which impact upon quality of life and the capacity to build relationships. Positive relationships, self-adaptability and positive affect have all been identified as important criteria for a full recovery beyond eating disorder symptom abatement [73], highlighting the importance of identifying and targeting these difficulties in treatment.

Negative temperament on the SNAP-Y was not significantly correlated with the eating disorder risk or the overcontrol composites of the EDI-3, despite mean scores for all three being high. This lack of association in the current study, particularly between the SNAP-Y and the EDI-3 overcontrol composite, suggests a general tendency towards negative affectivity may be a co-occurring but distinct factor to eating disorder and related psychopathology. The only EDI-3 composite significantly associated with negative temperament was the Ineffectiveness composite, which combines the low self-esteem and personal alienation scales. These findings may, in part, be due to the very broad nature of what the SNAP-Y negative temperament subscale is measuring. It assesses tendencies towards a wide range of general negative affectivity and distress which may be too non-specific to correlate with overcontrol as measured on the EDI-3. Further research is needed to understand the relationship between the broad concept of negative temperament, symptoms of restrictive eating disorders and other overcontrol related factors.

The observed relationship between overcontrol factors and eating disorder symptomatology also needs to be considered in the context of the specific group of adolescents in the current study. All participants in this study were diagnosed with a restrictive eating disorder, meaning generalisability of the findings to other types of eating disorders cannot be made from the current study. Given the differences in temperament and personality profiles (see Atiye et al. for review, 22), as well as differences in emotion regulation difficulties [74], across different eating disorders, it is possible that the observed relationship is specific to restrictive eating disorder presentations only. Further investigation is required.

The second aim of the study was to determine whether ITP with RO-DBT skills classes could lead to improvements in eating disorder symptoms, overcontrol related factors and the quality of relationships. The results described above indicate that ITP with RO-DBT skills classes led to significant improvements in physical health, eating disorder symptoms, and mood, with correction for multiple comparisons using the Benjamini-Hochberg procedure [72]. The programme also led to significant improvement in overcontrol factors like cognitive flexibility, reward responsiveness, emotional expression and openness to new pleasurable experiences. The results further indicate that adolescents experienced significant improvements in their relationship quality, reporting that they felt more comfortable in their relationships, prioritised them more, felt more connected to others and were less socially withdrawn.

The results also showed that temperament and obsessional traits remained largely unchanged. This is an expected finding given emerging evidence that personality traits and temperament show moderate to high stability from childhood and adolescence onwards [52, 75–78] and that temperament is presumably largely biologically based [53]. What does appear to be changeable is how the adolescents in this study coped with this. The pattern of results suggest ITP with RO-DBT may be providing adolescents the skills to manage temperamental overcontrol traits in more adaptive ways through the increased expression of their emotions, improved relationships and processing of rewards. All of which occurred alongside improvements in physical health and psychological symptoms linked with eating disorders.

The largest change observed in this study was on the suppression of emotional expression subscale of the emotion regulation questionnaire (ERQ-suppression; large effect size). This finding indicates that adolescents considered themselves to be less reliant on inhibiting their emotional expression to manage their emotions and more emotionally expressive by the end of treatment. Being more emotionally expressive in relationships is a core treatment target in RO-DBT and is hypothesised to be one of the main treatment mechanisms via which social connection and psychiatric symptomatology improves [35]. This finding suggests that the treatment may lead to changes in the domains RO-DBT directly intends to target.

Medium effect size changes were observed for level of social withdrawal and perceived improvements in the experience of pleasure, while the changes in eating disorder symptoms were only small. Similarly, changes of only small effect size were observed in depressive symptoms, attachment and relationship quality. It is possible that this may simply reflect ITP’s design and its brevity. ITP only aims to support adolescents and their families to reach a stage whereby further outpatient treatment can be sufficient and effective. ITP is not designed to support adolescents to reach a full recovery. Full recovery from a restrictive eating disorder is not expected within a programme with a mean duration of 13.4 weeks. Similarly, large changes in attachment and relationship quality are expected to take much longer than the duration of ITP. Future studies are needed to determine whether changes of the magnitude observed in this study are sufficient for the resumption of an effective and less costly outpatient treatment.

After correcting for multiple comparisons using the Benjamini-Hochberg procedure [72] the pattern of results remained largely unchanged. All significant changes observed in this study remained significant except for four subscales of the EDI-3, none of which were related to core eating disorder symptomatology (interpersonal alienation, asceticism, low self-esteem, interpersonal insecurity), and the Preoccupation subscale of the ASQ. After correction, significant changes were observed in the level of relationship quality (connectedness, withdrawal, comfort in and importance of attachment relationships), emotional expressiveness, reward sensitivity and flexibility; all characteristics targeted by RO-DBT.

Together these findings suggest that individuals enrolled in ITP with RO-DBT skills classes demonstrate significant improvements over a relatively short duration (mean duration 13.4 weeks) in both eating disorder and related symptoms as well as overcontrol characteristics. More than 70% of adolescents in this study, all of whom had not responded to or been appropriate for outpatient treatments, had a good or intermediate outcome at the end of ITP. Nevertheless, it is unclear whether symptom change was truly due to the RO-DBT components of the ITP programme, specifically, or other aspects of the programme. This will be an important focus of future research.

This study also shows that ITP can support severely ill adolescents with restrictive eating disorder to remain at home with very few requiring inpatient treatment either during or after ITP (4.6%). Rates of transfer and discharge to inpatient care can be difficult to compare across day programmes as they are likely influenced by individual programme factors, such as intensity, duration and staffing. However, rates of inpatient admissions in the current study are low compared to other published day programme studies which report between 13 and 35% of young people require an inpatient admission either during or after day programme treatment [8, 9, 20]. Prior to the introduction of RO-DBT into ITP, Simic et al. [8] published a case series of 105 adolescents who attended ITP between 2010 and 2015 with similar characteristics (mean age 15.5 years, range 11–18; 95.2% with AN; 95% female). They reported that 18% (*n* = 19) were referred for an inpatient admission during day programme treatment. This 2010–2015 case series evaluation [8] was completed before RO-DBT classes replaced Dialectical Behaviour Therapy groups in the programme, however no changes were made to the inpatient admission criteria across both study periods. The content of the remaining manualised therapy groups was identical in both case series. While this large reduction cannot be solely attributed to the introduction of RO-DBT skills classes, it was the only intentional change made to the programme. It would be useful for future research to determine whether specific treatment models are more effective than others in a day programme context.

This study has several important limitations. First, this was an uncontrolled case series making it difficult to draw conclusions about the specific contribution of RO-DBT to the outcomes. The design of the current study does not allow for any investigation into how different factors interact during day programme treatment or whether improvements in overcontrol coping lead to or are the result of improvement in other areas. As all measures used in this study were self-report, there is also potential that shared method bias is impacting the high level of correlations amongst variables. Future research studies are needed to replicate the current findings and explore effectiveness of specific components and mechanisms of change within day programme treatments.

Second, this was not a study of either pure RO-DBT or of all RO-DBT components. RO-DBT in its original form covers 30 skills classes plus individual sessions. Given the mean length of attendance was 13.4 weeks, even with two RO-DBT skills classes per week, it is possible that not enough time passed to practice skills and observe more meaningful changes. Adolescents also did not receive individual RO-DBT sessions and they received a range of other therapeutic interventions that are likely to have contributed to treatment outcomes.

Third, being a case series in a clinical service, there were inconsistencies in data collection and missing data. Additionally, some of the measures used were not validated with adolescents and were included in the absence of such measures being available (e.g. FFOCI). It is also important to note that some improvements observed in this study, have not been measured before in adolescent eating disorder population, leaving open for consideration that these changes could occur following any day programme if the same factors were measured.

Additional limitations include the limited generalizability of the findings to more diverse groups, people with less severe eating disorder symptoms, across different treatment settings and different treatment format (individual, family, group etc). In addition, when considering the clinical impact of ITP with RO-DBT, an important future direction would include efforts to obtain post-treatment follow-up data to determine the extent to which the reported symptom improvements persist after treatment. Improvements while in treatment are promising; however, the longer-term efficacy of interventions would provide further support for this intervention approach.

## Conclusions

This case series of a sizeable sample of all consecutive admissions to a day programme over four years, offers preliminary support for the effectiveness of RO-DBT informed day programme treatment for restrictive eating disorders. The results also provide tentative support for an alternate way of conceptualising treatment and recovery from restrictive eating disorders for adolescents in a day programme setting. This study evaluates outcomes with a different lens in a field that struggles to find consensus on defining recovery beyond weight gain and eating disorder symptom reduction [73, 79]. By showing that a day programme that includes RO-DBT classes can facilitate significant changes in the quality of relationships and the way bio-temperamental factors are managed, the current findings may have important and meaningful implications when considering broader psychological wellbeing, functioning and quality of life; all factors that require much more focus when considering recovery from an eating disorder.

## Data Availability

Data generated and analysed for this study are not available to be shared. Audit data are not permitted to be shared by the South London and Maudsley Child and Adolescent audit committee.

## Abbreviations

AN: Anorexia Nervosa
ASQ: Attachment Styles Questionnaire
CBT: Cognitive Behavioural Therapy
CRT: Cognitive Remediation Therapy
DBT: Dialectical Behaviour Therapy
EDI-3: Eating Disorder Inventory – 3rd Edition
ERQ: Emotion Regulation Questionnaire
FFOCI-SF: Five Factor Obsessive Compulsive Inventory – Short Form
ITP: Intensive Treatment Programme
MFQ: Moods and Feelings Questionnaire
MCCAED: Maudsley Centre for Child and Adolescent Eating Disorders
RO-DBT: Radically Open Dialectical Behaviour Therapy
SCS-R: Social Connectedness Scale – Revised
SNAP-Y: Schedule of Non-Adaptive and Adaptive Personality for Youth
TEPS: Temporal Experience of Pleasure
TEPS-CON: Temporal Experience of Pleasure – Consummatory
TEPS-ANT: Temporal Experience of Pleasure – Anticipatory subscale
WS-YSR: Youth Self Report – Withdrawal subscale
%mBMI: Percentage of median body mass index
